# Pulmonary Emphysema

**Published:** 1852-08

**Authors:** 


### RECORD OF MEDICAL SCIENCE.

### PATHOLOGY AND PRACTICE OF MEDICINE.

*Pulmonary Emphysema.*—Dr. Seller read a paper entitled, "On the Production of Vesicular Emphysema in the Lungs, as Explicable by the Principles of Pneumatics."

The object of this paper was to show that the act of expiration must be essentially the cause of vesicular emphysema, in so far as it is a mechanical effect, in opposition to the view upheld by many recent authorities, under which it is regarded as a result of forcible inspiration.

Founding on the acknowledged belief, that in inspiration the air enters each air-cell on the principle of suction, the author concluded, that the inspired air could only become a cause of unusual distension to any portion of the air-cells, if it were found that the inspiratory muscular forces were capable, under given circumstances, of becoming developed to an extent sufficient to overcome a large proportion of the atmospheric pressure exerted on the outer surface of the walls of the chest—a pressure amounting, on a moderate computation, to 10,000 pounds.

To the view adopted by Dr. William T. Gairdner, in his recent papers on "Bronchitis and Bronchial Obstruction," Dr. S. devoted a particular consideration. He represented Dr. Gairdner's view as an assumption supported only by a limited number of facts, to the effect, that vesicular emphysema never occurs unless the dilatability of the lung, by the collapse of portions of its substance, has become less than the expansibility of the chest under the inspiratory forces. Dr. S. described Dr. G.'s view as highly ingenious, and much less at variance with the obvious principles of pneumatics than the rest of the views which ascribe vesicular emphysema to inspiration; but he nevertheless regarded it as untenable, for the following reasons :—1. Because there is great reason to doubt if collapse of a portion or portions of the lungs be uniformly a concomitant of vesicular emphysema; 2. Because it is an unsupported assumption that the chest can expand beyond the present limits to which the lungs can dilate, the contrary being plainly the general rule; 3. Because, were vesicular emphysema so produced, there could be no partial form of the disease, but every part of the lung of one side unaffected with collapse would exhibit a similar state of pathological alteration; 4. Because, though no unusual inspiratory force, in the case supposed, would be requisite to overcome atmospheric pressure on the exterior of the chest, since, could the enlargement occur, it might take place no faster than the air could enter from without; yet an unestimated additional inspiratory force would be necessary to overcome the resistance of the aggregate of the walls of all the still pervious air-cells, in each inspiration by which a distension beyond their greatest natural magnitude was effected —the possibility of the exertion of such a force being an unsupported assumption; 5. Because, it is not shown, in the cases of vesicular emphysema founded on by Dr. Gairdner, that the aggregate of the distension of the air-cells is in exact correspondence, as to volume, with the aggregate of the decrements which the collapsed portions of the lung have suffered, which should hold, were this view correct.

In support of the view, that expiration is the mechanical cause of vesicular emphysema, Dr. S. referred to forcible compression of the pulmonary air-bag during acts of expiration, great in proportion to the rapidity with which the expiatory forces are exerted, and to the amount of impediment offered at the moment to the free egress of the air by the windpipe; and also to those occasions when, after a very full inspiration, the powerful muscles of expiration violently compress the abdomen and thorax at the same time that the larynx is forcibly closed, so that the air within the lungs is strongly condensed before it is permitted to escape outwards, by the relaxation of the orifice of the larynx. He referred to the frequency of vesicular emphysema in draught-horses, in which it is plainly produced by acts of the latter kind. In proof that pressure of this kind could exercise a distending force on the air-cells when it took place unequally, he referred to the bursting of a bladder by a weight placed upon it, and to the bursting of a paper bag of air when struck at one end. He admitted, however, that the double pressure from without and from within at the same moment, may so far injure the vital texture of the walls of the air-cells, by the compression of the blood-vessels, as to render the distending force more effectual than it otherwise would be.

Dr. W. T. Gairdner said, that, notwithstanding the arguments advanced by Dr. Seller, he was still of opinion that the inspiration-theory under the modification which he had advanced in his papers on bronchitis, was the only one which could on mechanical principles account for the production of emphysema. He could not at that meeting enter into the discussion of the exceedingly elaborate paper just read, but he would make one or two observations, and endeavor, at the same time, to place his own view in a simple and clear form before the members. But first, as to the expiration-theories of emphysema in general, he was still at a loss to understand how the force of expiration could ever become a cause of dilatation, either of the whole pulmonary sac, or of a portion of its vesicle. Expiration was essentially a *compressing* force, acting from without, and tending to *contraction* of the air-vesicles; and although the effect of this force might to a certain extent be counteracted or neutralised, when the glottis was closed, by counter-pressure from within, the amount of this internal pressure was always exactly proportionate to the external, and could, therefore, never act so as to produce distension. The case of the air-filled bladder burst by external pressure, did not, in Dr. G.'s opinion, at all bear upon the question; for, in the first place, the bladder in such circumstances bursts because it is compressed in only one direction, and allowed to expand indefinitely in others, whereas the lung is guarded on all sides by the thoracic parietes, which, in the physiological act of coughing, or other impulsive expiratory acts, prevent its air-cells from being subjected to any kind of distortion of form, such as occurs under the one-sided pressure which produces bursting of the bladder. But, further, the blown bladder, although burst by external pressure, is really never distended, but on the contrary diminished in volume; whereas no one can doubt that in emphysema the air-cells are not so much altered in form as subjected to great distension. The blown bladder is flattened by the force which bursts it; the emphysematous air-cells are permanently expanded, and retain the rounded appearance, even when many of them have been fused into one large bulla by partial destruction of their walls. But, supposing it granted that the expiatory forces might, under peculiar circumstances, act so as to produce partial compression and disorganization of individual air-cells, this would not account for the cases where inordinate distension of the air-cells was observed over a large extent of the lung; still less for the alleged cases of universal emphysema, which had been advanced in opposition to his (Dr. G.'s) theory. The view he (Dr. G.) took of the production of emphysema was this:—Two complex forces acted mechanically on the lung in respiration; the one, that of expiration, a compressing and contracting force as regards the pulmonary air-cells; the other, that of inspiration, an expanding and dilating force. Independently of the arguments already used, he thought it natural to ascribe emphysema, in so far as this was a mechanical lesion, to the action of the latter or dilating force, in preference to the former. It was not difficult to conceive that, as the forces of inspiration have the power, physiologically, to distend their air-cells *up to* their normal maximum, so they might, under certain pathological circumstances, have the power of distending certain air-cells *beyond* their normal maximum. This was what had been assumed to take place by all the advocates of the inspiration theory of emphysema; none of whom, however, had, in Dr. G.'s opinion, correctly generalized the condition of emphysema, or stated the law of its production. In fact, the lung was accurately adapted by nature, in size and form, to the case in which it was enclosed, and the alternate expansion and contraction of which it followed, in consequence of mechanical laws which were understood by every one. No amount of inspiratory power could, in the physiological state, dilate any portion of the lung inordinately, because the thoracic case in which the lung was enclosed was not dilated beyond a certain amount, and the lung was adapted by nature to bear dilation to that amount without injury. But let any portion of the lung be rendered undilatable by disease, *being at the same time diminished in volume,* and it then became possible for the chest to expand so as inordinately to dilate the remaining pervious air-cells, which would tend to occupy the space forfeited by those which were diminished in bulk. Contraction, collapse, or atrophy, therefore, of some portions of the lung, from disease, with inordinate expansion of the sounder portions under violent acts of inspiration, were the conditions under which emphysema was produced. In writing at large on the subject of bronchitis and its consequences, Dr. G. said, he had been at some pains to describe the variety of these lesions observed in the dead body, and to demonstrate by an analysis of cases of emphysema, the coincidence of that affection with the states of the lung leading to partially diminished volume. He had also fully explained the sources of collapse and atrophy of the lung, and directed attention to the great frequency of these affections, and to their connection with, and dependence upon, obstruction of the bronchi in bronchitis and other diseased states. He was, therefore, prepared to state that his theory of emphysema was the fruit of an extensive observation of facts, and he believed it would be found universally applicable, to the exclusion of the expiration-theories, and all the inspiration-theories not founded on the idea of partially diminished bulk in the lung, as the essential cause of emphysema. Other circumstances in the natural history of emphysema were perfectly explained by this theory; as, for instance, its ordinary seat in the anterior edges, the parts least subject to other diseased conditions; its frequency in connection with retrograde or cicatrising tubercle, and its comparative rarity in connection with advancing tubercular disease, and generally with all states of the lung in which large excavations, with flaccid dilatable walls, took the place of the pulmonary air-cells in maintaining the full volume of the lung in the act of inspiration. For the fuller developement of his views, Dr. Gairdner referred to his memoir on bronchitis, and expressed himself much gratified with the intelligent and careful consideration which Dr. Seller had thought it worth while to bestow on his (Dr. G.'s) labors.

Dr. Alison remarked that he was disposed to agree generally with Dr. Seller in believing emphysema to be produced in expiration. He had never been able to understand what force, adequate to the rupture of the walls of the cells, could be supposed to be called into play by any effort of *inspiration*. He did not think that emphysema of the lung was *always* determined by other lesions, as said by Dr. Gairdner; and thought he had seen cases of emphysema affecting the whole lung, at least unattended by such condensation of any part of the lung, as to render it impervious to the air.

Mr. G. Glover commenced by remarking that, in the present transition state of medical science, he was glad to find the author of the paper appealing to acknowledged principles in physics. It was dangerous to proceed practically upon an imperfect knowledge of a few first principles in science; and when applying the principles of pneumatics as "explicable of vesicular emphysema," our ideas must be clear and precise, and the application correct;—not attributing to causes effects for which they are quite inadequate. The unscientific term "suction," used in the paper, was vague, and often meant nothing, at least nothing upon which correct reasoning could be founded. The enormous pressure attributed to the tendency to a vacuum ("suction") in the chest during tranquil breathing, was greatly overrated. And to show the small amount of the force, he suggested to Dr. Seller the simple experiment of putting one end of a glass tube in water, and the other end in his mouth, during ordinary breathing, when he would observe the water scarcely rising *one inch* in the tube during each inspiration, instead of thirty-three feet, which would be required to represent about fifteen pounds of pressure on every square inch of surface. With regard to Dr. Seller's observation on the bursting of a bladder and a child's paper bag, he stated, that while the paper bag would be easily burst by a sudden percussion, the bladder containing air would sustain hundreds of pounds of pressure before bursting. It is a fundamental principle in pneumatics, that pressure exerted on a fluid must be equal, and in all directions; so that no part of a bladder containing air, and subjected to pressure, could be unequally pressed upon. The bursting pressure would be equally diffused over every part of the surface of the bladder. As an illustration of the principle of equal pressure in fluids, he gave the instance of the early development of the embryo in the uterus, where the most delicate structure floating in a fluid is not ruptured by the violent motions of the mother, or by external violence. He had listened attentively to the paper, but was not prepared to support either theory, regarding the production of vesicular emphysema by inspiration or expiration. If produced by inspiration or expiration, it would be of more frequent occurrence. At the same time, in case of asthma, he would feel more inclined to attribute the production or increase of emphysema to the long and laborious efforts of expiration than the short and easy inspirations. He mentioned that, some days before, he had opened the body of an asthmatic female, where the whole lungs appeared to be emphysematous; and when placed in water they floated very lightly on the surface. Drs. Keiller and Menzies were present at the dissection.

Dr. Alexander Wood was of opinion, that in the discussion, in the anxiety to attend to physical laws, vital laws had been too much overlooked. The natural tendency of every yielding tube was to become stretched when pressed by air on its internal surface. In health the air-cells of the lungs resisted this by their vital elasticity. The tendency of all the diseases with which emphysema was associated was to destroy this elasticity. He doubted the alleged fact on which the new theory was founded—that some diminution in the size of the lungs was found in every case of emphysema. He believed that the forcible entrance of air into the lungs could produce emphysema, as was witnessed as the sequel of too zealous efforts to resuscitate still-born children. He believed that impediments to the exit of free air did the same, or else how was emphysema produced in hooping cough? Not surely by the impeded inspiration, but by the impossibility of the air cells getting rid of their contents; while, at the same time, the collapsing lung and falling down parietes forcibly compressed it.

Dr. Strachan, of Dollar, in illustration of the applicability of the inspiration-theories to the production of emphysema, referred to the expansion and bursting of a partially air-filled bladder under the receiver of an air-pump, the exhaustion of the receiver producing on the walls of the bladder a similar effect to that exerted by the expansion of the thoracic parietes on the lung.

Dr. Bennet observed that, with respect to vital and physical properties, he considered that the former were so called simply because they could not, in the present state of science, be explained by physical laws, and were peculiar to living beings. The present tendency of physiology was to cultivate physics and chemistry; and in proportion as these explained what was formerly unknown, the so-called vital phenomena diminished in number. No doubt some of these latter, such as contractility in muscle, sensibility in nerve, reproduction, and so on, had not yet been brought into the domain of physics, and hence why these must still be considered as purely vital phenomena. With regard to emphysema, however, the case was different; and it appeared to him that its production, like the phenomena of respiration, was, to a certain extent, explicable by physical laws. In such a case he was unable to understand what Dr. Wood meant by vital, as distinguished from physical resistance of the lung. It appeared to him clear, that if, in the healthy condition, the lungs completely filled the thoracic cavity, and that if, when diseased, a portion of their structure was collapsed, that the sound parts would be expanded by the pressure of the atmosphere, and fill up the vacant space. This was Dr. Gairdner's theory. It had indeed been urged, that the overdistended, or emphysematous pulmonary tissue, occurred without such collapse having been seen. But he considered this to be an example of what frequently occurred in pathological investigation, namely, that such partial collapse of a lung might not be seen, because not looked for. Let a lesion be once carefully described, and the attention of observers directed to it, and a morbid appearance formerly unknown subsequently became very common. This he believed to be the case with partial collapse and condensations of the lung as a cause of emphysema. He had himself remarked the frequency of emphysema in conjunction with certain diseases producing partial contraction and condensation of the lung, as in chronic phthsis. The same fact had been observed by Dr. Ramage, who, in consequence, had erroneously considered that the production of emphysema was the natural cure of tubercular caverns. He also agreed with Dr. Gairdner, as to inspiration being the act of real difficulty in cases of emphysema. The prolonged expiration was principally owing to the impaired elasticity of the pulmonary tissue itself, whereby the air-vesicles were not compressed, and the chief mechanical force during the expiratory act more or less impeded.

Dr. W. T. Gairdner observed, that he scarcely required to add one word in explanation to what had fallen from Dr. Bennet, with which he entirely agreed so far as the subject of emphysema was concerned. He would, however, remark, that there were three different forms of atrophic lesion, which might give rise to emphysema: the simple mechanical collapse of the lung; the simple chronic atrophy, resulting from that collapse; and the various kinds of atrophy with induration, resulting from pneumonia, tubercle, etc. The last could not be overlooked with moderate attention; but the first might easily be neglected by a superficial observer; and the second, having mostly negative characters, was almost sure to be overlooked by any one, however careful, who came unprepared to the investigation. Since he had given particular attention to the development of emphysema, however, Dr. G. had not found a single instance of that lesion in which he had not been able to demonstrate its connection with one or other form of atrophic disease.—*Trans. Med. Chir. Soc. in London Monthly Journal, May* 1852.

